# Assessment of corn starch as substitute for agarose in DNA gel electrophoresis

**DOI:** 10.1186/s13104-021-05483-1

**Published:** 2021-02-25

**Authors:** Francis Tanam Djankpa, Gideon Akuamoah Wiafe, Bernard Ntim Boateng, Korantema Mawuena Tsegah, Samuel Essien-Baidoo, Mark Bilinyi Ulanja, Kwame Ofori Affram, Abdala Mumuni Ussif, Desmond Owusu Agyeman, Gabriel Asante

**Affiliations:** 1 Department of Physiology, School of Medical Sciences, College of Health and Allied Sciences, University of Cape Coast, Cape Coast, Ghana; 2 Department of Biomedical Sciences, School of Allied Health Sciences, College of Health and Allied Sciences, University of Cape Coast, Cape Coast, Ghana; 3 Department of Medical Laboratory Sciences, School of Allied Health Sciences, College of Health and Allied Sciences, University of Cape Coast, Cape Coast, Ghana; 4 Department of Internal Medicine, University of Nevada, Reno School of Medicine, 1155 Mill St, Reno, NV 89502 USA; 5 Piedmont Athens Regional Medical Center, Graduate Medical Education, Athens, GA USA; 6 Department of Forensic Sciences, School of Biological Sciences, University of Cape Coast, Cape Coast, Ghana

**Keywords:** Electrophoresis, Gel, DNA, Corn, Starch, Agarose

## Abstract

**Objective:**

The use of agarose in nucleic acid electrophoresis is the gold standard. However, agarose is very expensive and not readily available in resource limited developing countries like Ghana. Hence, finding a more affordable and readily available alternative to agarose will be a major boost to molecular research in developing countries. This study was aimed at investigating the use of corn starch as a potential substitute for agarose in DNA gel electrophoresis.

**Results:**

Genomic deoxyribonucleic acid (DNA) extracted from *Plasmodium falciparum* and primers were obtained from the West African Centre for Cell Biology of Infectious Pathogens and amplified using polymerase chain reaction. The amplicon was run on agarose gel to ascertain the molecular weight (as a positive control). When visualized under both blue light and ultraviolet light, the DNA and ladder showed clear and clean bands with the expected molecular weight. Corn starch was then modified with sodium borate buffer, casted into a gel and used to run the same DNA sample. Our findings indicated that similar to agarose, the DNA sample and ladder migrated successfully through the modified starch gel but no bands were visible when visualized under blue and ultra-violet light.

## Introduction

One of the most important techniques employed in molecular biology is gel electrophoresis, the separation of charged molecules such as nucleic acids and proteins in a stationary colloidal medium by passing an electric current through it [1]. The popularity and standard use of agarose gels as electrophoretic medium stems from many advantages that agarose offers. Chiefly, that gels are easy to cast [2], are suitable for separating large and moderately-sized DNA molecules [3], have wide separation range [4] and provide a nontoxic gel medium [3]. However, agarose gels produce poor band resolution and are unsuitable for samples of low molecular weight [3]. These disadvantages may be remedied using polyacrylamide gels. Polyacrylamide gels also have disadvantages. Acrylamide, from which the gel is prepared, is toxic and the gels are fragile, difficult to handle and tedious to work with [3]. The cost of both agarose and acrylamide is disapprovingly high and constitute a hindrance to molecular studies in resource-poor laboratories of developing countries. An exploration of other suitable electrophoretic media is therefore needed to address this challenge.

Prior to the emergence of agarose as the dominant electrophoretic medium in the mid-to-late 1960s, materials such as starch and agar were also in popular use [5]. Starch as an electrophoretic medium has sadly fallen out of popular use in modern science. Importantly, starch is abundant and inexpensive in sub-Saharan Africa, which could be great assets for the scientific community in that region.

We therefore assessed the usefulness of corn starch as a substitute for agarose in DNA gel electrophoresis.

## Main text

### Methods

#### Amplification of histidine rich protein-II (Hrp-II) of *Plasmodium falciparum*

The genomic DNA was amplified through polymerase chain reaction (PCR). The DNA amplicon was run on agarose gel to ascertain the molecular weight. Thereafter, the amplicon was also run on modified corn starch gel for comparison.

The PCR was run using the New England Biolab (NEB) PCR master mix. The PCR reaction mixture was set to a total volume of 25 µl. The tubes were transferred into the thermal cycler (GeneAmp^®^ PCR System 9700), set according to NEB guidelines. Briefly, an initial denaturation process at 94 ºC for 30 s, a 30-cycle process involving three different temperatures and time readings (94 ºC for 30 s, 55 °C for 30 s and 68 ºC for 50 s), a final extension process at 68 ºC for 5 min and a hold at 4 °C after the process was complete.

#### Preparation of 1 L of 1X TAE buffer from 50X stock

A volume of 20 ml of 50X TAE (tris–acetate EDTA (Ethylenediaminetetraacetic Acid)) was measured into a 1 L beaker. It was topped up with 980 ml of distilled water up to the 1 L mark to obtain a working solution of 1 ×.

#### Preparation of 0.9% agarose gel

An amount of 0.9 g of agarose powder was weighed and transferred into 100 ml of the prepared TAE. It was stirred gently and microwaved for 60 s initially, and 20 s subsequently until the solution was clear. The solution was allowed to cool for 5 min and 30 µl of DNA staining dye (Apex safe DNA gel stain) was pipetted into the agarose solution. The solution was stirred gently and poured into the gel cast. A fifteen (15) well comb was inserted and the gel was allowed to solidify. The comb was gently removed and the gel placed into a horizontal gel tank (Cleaver Scientific). The 1 × TAE solution was poured into the gel tank to the maximum mark.

#### Loading samples into agarose gel

Twenty microliters (20 µl) of 1 kb DNA ladder (NEB) was pipetted into the first (1st), ninth (9th) and twelfth (12th) wells. The Apex safe DNA loading dye (5X) was added to all samples to obtain a final concentration of 1X. Twelve microliters (12 µl) of the DNA and dye mixture was loaded into the fourth (4th) and seventh (7th) wells and electrophoresis was run at 80 V and 300 Watts for 2 h. The gel was removed and viewed for band formation using a blue light box (Accuris Instruments™ Smartdoc™).

#### Corn starch gel preparation

The industrial corn starch was modified and used to prepare the gel. A literature search found no clear protocol to suit our purpose. We therefore modified and adjusted protocols previously described [6]. Hence, we used a novel protocol for making starch gel by modifying previous ones. Thirty-six grams (36 g) of the corn starch was weighed. Boric acid and sodium hydroxide buffer were prepared by weighing 1.855 g of boric acid into a 1 L beaker which contained 200 ml of distilled water and 0.48 g of NaOH was added to the solution. The solution was topped up with distilled water to the 1 L mark. The weighed corn starch was poured into 200 ml of prepared boric acid and NaOH buffer. It was stirred very well and allowed to stand in water bath for 30 min at 50 °C. The supernatant was discarded and the sediment retained. Thirty (30) ml of distilled water was added to the sediment and mixed carefully. The sediment was allowed to settle and the supernatant, gently poured out. It was dried overnight at 70 °C in an incubator. After drying, the starch was ground into a smooth powder using a pestle and mortar. An amount of 12 g of the modified corn starch was weighed. Hundred (100) ml of TAE buffer was measured into a glass beaker and boiled on a hot plate with constant stirring. The modified starch was added to the boiling TAE buffer and stirred carefully for a homogenous mixture. The mixture was allowed to cool down for about 3 min and then thirty (30) µl of Apex DNA gel stain was pipetted into the mixture and stirred to mix evenly. The cooled starch was poured into the horizontal gel cast tray with comb inserted and allowed to stand overnight at room temperature to dry gradually and attain a firm texture.

#### Loading samples into corn starch gel

Twenty microliters (20 µl) of 1 kb DNA ladder (NEB) was pipetted into the first (1st), ninth (9th) and twelfth (12th) wells. We added 5X Apex safe DNA loading dye to all samples to obtain a final concentration of 1X. Twelve microliters (12 µl) of the DNA and dye mixture was loaded into the fourth (4th) and seventh (7th) wells and electrophoresis was run at 80 V and 300 Watts for 2 h. The starch gel was removed and viewed for band formation using a blue light box (Accuris Instruments™ Smartdoc™).

### Results and discussion

**Fig. 1:**
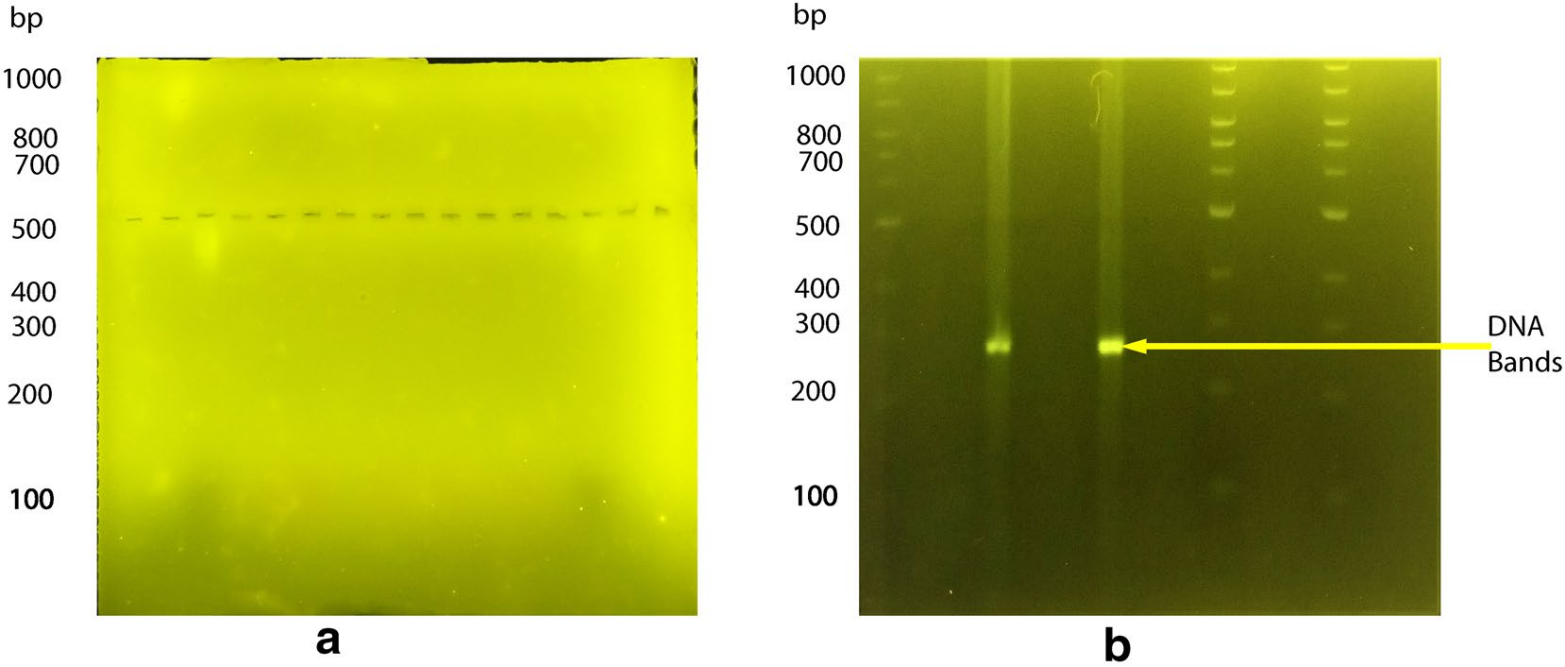
Comparison of gels viewed under blue light. **a** Corn starch gel viewed under blue light with no bands seen. **b** Agarose gel viewed under blue light showing DNA bands. The first and the last 2 lanes show the 1 kb DNA ladder whereas the middle samples show the DNA bands around the expected molecular weight of about 300 base pairs

**Fig. 2:**
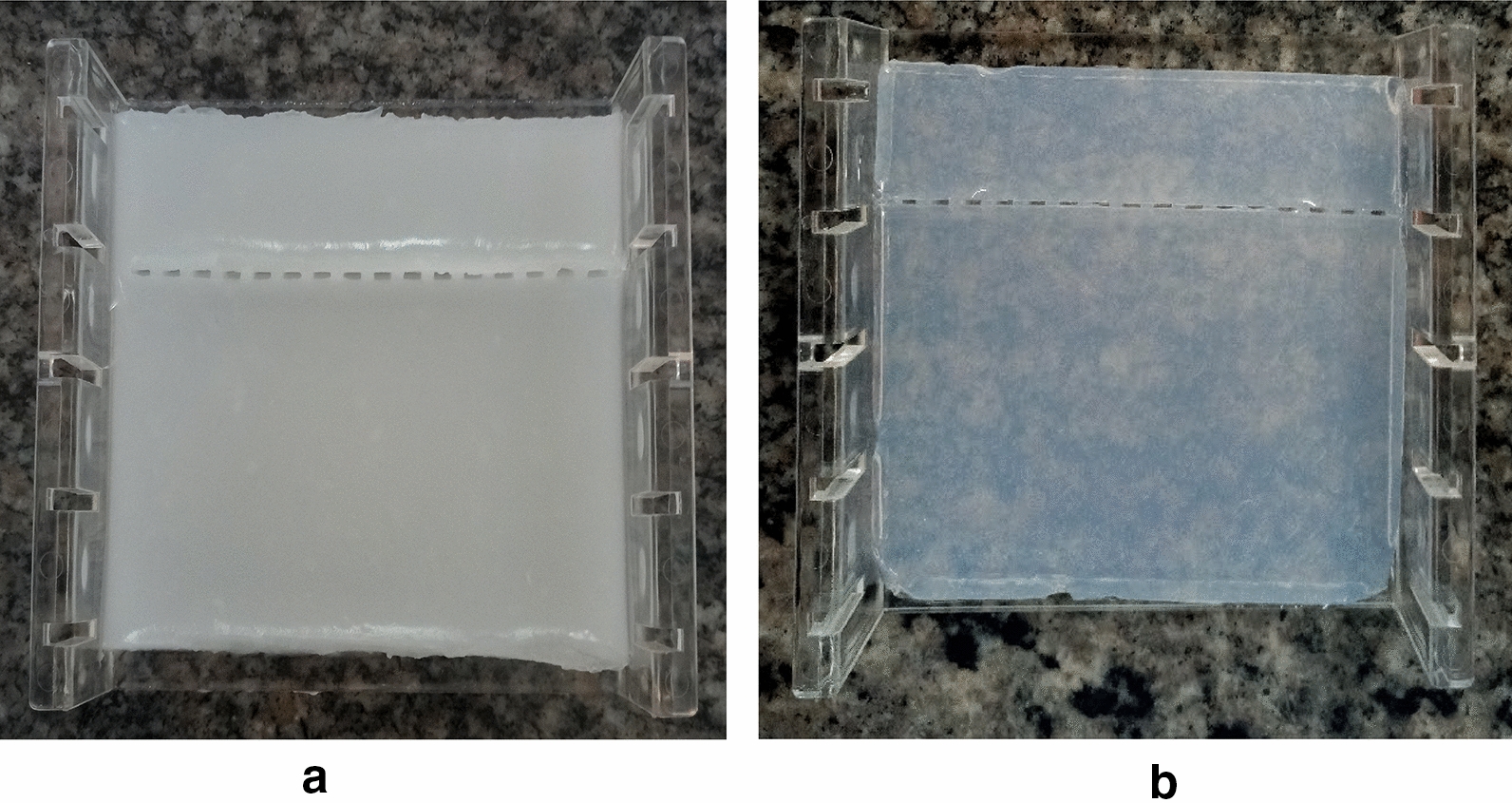
Comparison of modified corn starch gel and agarose gel. Wells were clearly formed in both gels. **a** The corn starch gel appeared cloudy and translucent. It was fragile. **b** Agarose gel was transparent, firm and easy to handle

**Fig. 3:**
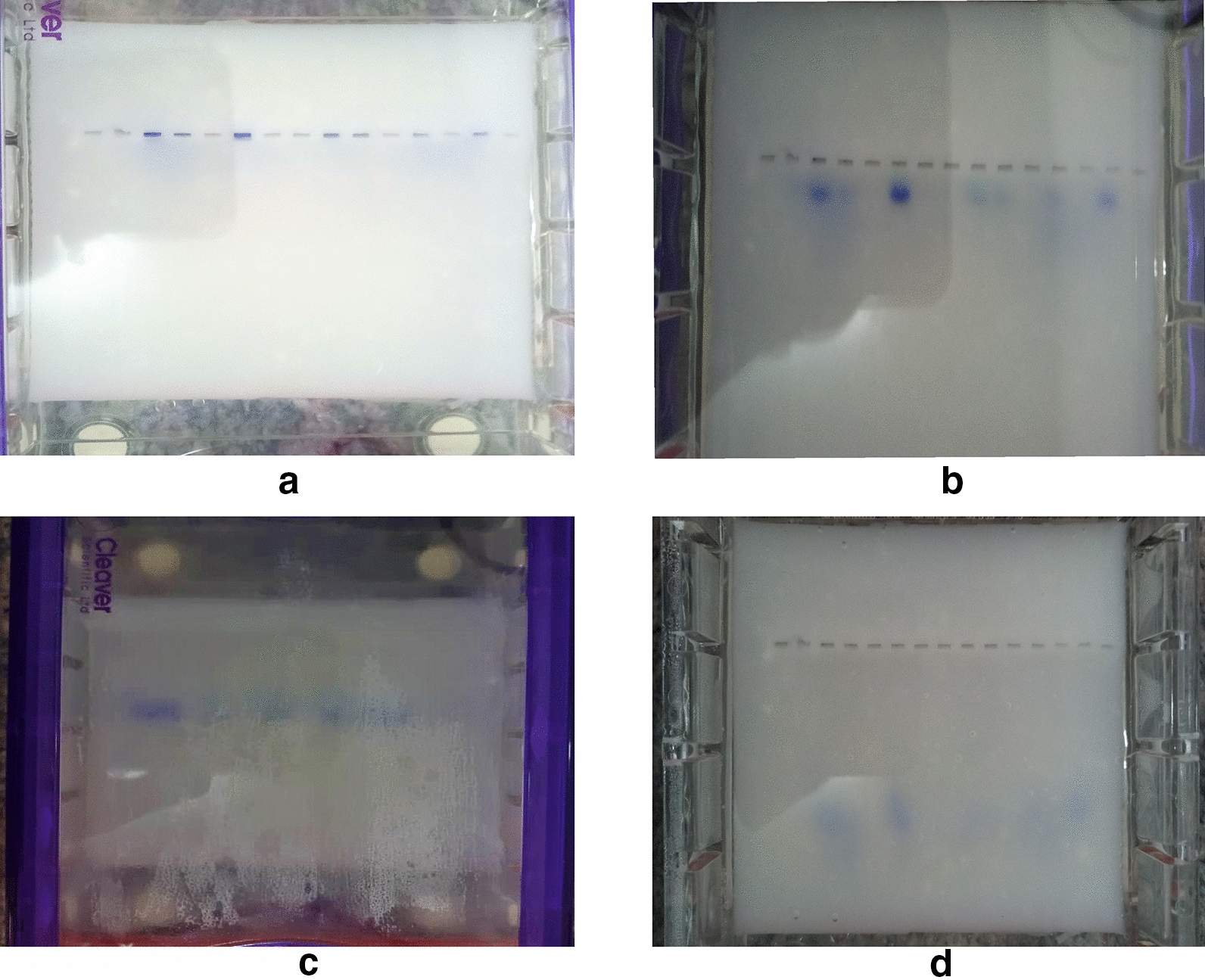
Migration of loaded DNA ladder (DL) and DNA sample (DS) on corn starch gel. **a** Shows the loaded samples (DNA + Apex Safe DNA loading dye), and the 1 kb DNA ladder (DL) on the starch gel at the onset. **b** Shows a migration of samples and DL 10 min after we started running the gel. **c** Shows migration of samples and DL at 30 min after we started running the gel. **d** Shows migration of samples and DL at 90 min after we started running the gel

Starch is composed of two large α-linked glucose-containing polymers namely the smaller and linear (amylose), and the very large and highly branched (amylopectin) [7], whereas agarose consists of repeated residues of 1,3-linked β-D-galactopyranose and 1,4-linked 3,6-anhydro-α-L-galactopyranose [8]. Agarose gel electrophoresis was done as a positive control to corn starch gel electrophoresis. The genomic DNA (Hrp-II gene), was found to have a molecular weight of 300-bp as expected (Fig. 1b). The starch gel was prepared over a 24-h period and yielded quiet satisfactory results for electrophoretic use. These observations revealed that corn starch under appropriate and carefully prepared conditions has the potential to be used for DNA gel electrophoresis especially in resource-limited countries. The starch gel prepared from the modified starch appeared a little cloudy and translucent initially with some opacity as it cooled down compared with that of agarose. The wells into which the samples were loaded were clearly formed providing some proof of the gelling properties of the corn starch (Fig. 2). The DNA samples were seen migrating clearly through the corn starch gel at different times throughout the electrophoresis i.e. after 10 min (Fig. 3b), 30 min (Fig. 3c) and 90 min (Fig. 3d). Branch chain lengths of amylopectin and their distributions play a major role in affecting the gelatinization temperature of starch. Without other structural differences, such as phosphate monoester contents, starch which contains longer branch chain lengths displays a higher gelatinization temperature and forms a stronger gel [9]. According to Meyer and colleagues [10], starch paste without added amylose would yield a highly viscous liquid and this was observed in our experiment hence the need for starch modification before gelling. The use of a higher concentration of starch without addition of amylose, on the other hand, may lead to the formation of a solid gel [10].

During our experiment, the migration of DNA samples and the ladder occurred through the corn starch gel as shown in Fig. 3 but no DNA bands were seen when visualized using the blue light box and ultra violet light box. The absence of DNA bands in the corn starch suggests two things; either the cross bridges between the DNA and the DNA staining dye were not formed due to the structure of the starch gel or formed but were not visible due to the level of opacity of the starch gel. Cross bridges are usually formed by a cross reaction between the DNA staining dye (Apex safe DNA gel stain) and the DNA molecules and these become visible under blue light or ultra violet light. This was possibly due to differences in reactivity between starch and the DNA staining dye. Secondly, the bands could have been formed but were invisible due to the level of opacity of the corn starch gel. A similar experiment was performed by Smithies [11] where he showed the group variations in the serum proteins of normal human adults using starch as a gel for zone electrophoresis. He found that most of the new components demonstrated by the starch gel method are derived from α2-globulin, which proves to be a complex mixture of many different proteins migrating in the starch gels ahead of β-globulin and some behind. Similarly, Bernfeld and colleagues [12] also conducted an experiment in which they showed the separation of protein by zone electrophoresis on a starch gel. The use of starch gel to separate proteins by these authors give a hope for future studies that DNA can be separated using starch gel. We therefore recommend that further work be done on the optimization of starch gel in DNA gel electrophoresis.

## Limitations

We had few limitations in this research. The starch gel we prepared was optimized at 12% versus but we were compelled to use 0.9% for the agarose gel for comparison. Because of the large size of our cast and the nature of our protocols, we needed a higher quantity of starch to fill it up to ensure uniformity and equal thickness of our gel. Ideally, we should have match up the agarose to 12% but our DNA amplicon size was only suitable for low percentage gel of 0.9–2%. Due to limitation in resources we got the DNA and primers as gift from another laboratory and run the PCR to get our amplicon which was suitable for a low molecular weight gel. The disparity in molecular weight between starch and agarose gel could have accounted for the absence of bands on the starch gel. Although our research does not show any positive results, we do not rule out the possibility of starch having the potential to replace agarose in DNA gel electrophoresis in resource limited areas. We advocate for further research and further optimization of our protocols to arrive at a definite conclusion. We are very pleased to announce here that our modified corn starch protocol has produced some positive results leading to the formation of a solid and stable starch gel which clearly allowed the migration of both DNA and the 1 kb ladder. Here we wish to resound a take home message that based on our preliminary findings we believe that corn starch has the potential to replace agarose. The DNA sample and ladder showed clear migration through the corn starch which is great sign of potential breakthrough in the near future. Our findings though preliminary are reproducible and can also be optimized when given more resources.

## Data Availability

The data and material used during the current study are available from the corresponding author on reasonable request.

## Abbreviations

DNA: Deoxyribonucleic acid
Hrp-II: Histidine rich protein II
PCR: Polymerase chain reaction
NEB: New England Biolabs
EDTA: Ethylenediaminetetraacetic Acid
TAE: Tris–Acetate EDTA
NaOH: Sodium hydroxide
DRIC: Directorate of research innovation and consultancy
